# Geno-toxic risk assessment of locally produced bovine pericardium (LYOLEMB) for guided tissue regeneration

**DOI:** 10.6026/9732063002001489

**Published:** 2024-11-05

**Authors:** Abdo Mohammed Mohammed Abdulrazzaq, Sultan Alanazi, Ali M. Alyami, Abdullah Awad Alshehri, Turki Fahad Al Mansour, Yazeed Saleh AL-Ghwaynim

**Affiliations:** 1 Department of Preventive Dental Science, Faculty of Dentistry, Najran University, Najran, Saudi Arabia; 2 Faculty of Dentistry, Najran University, Najran, Saudi Arabia

**Keywords:** Ames test, LYOLEMB, safe, GTR, analysis

## Abstract

This study evaluated the genotoxic risk of locally produced bovine pericardium (LYOLEMB) as a guided tissue regeneration (GTR) material for advanced periodontal disease using the Ames test with metabolic activation (a sodium phosphate buffer). Mutagenic effects on Salmonella typhimurium strains TA 98, TA 1537, TA 100 and TA 1535 at concentrations ranging from 0.3125 mg/plate to 5 mg/plate showed no significant genotoxicity, with revertant counts remaining below twice that of the control. Statistical significance was observed near p ≤ 0.05 at certain concentrations, confirming LYOLEMB's non-mutagenic, biocompatible and safe use in periodontal therapy.

## Background:

The concept of GTR was first proposed by Melcher, who theorized that cell the types of cells repopulating the root surface post-surgery dictate the nature of attachment healing, this hypothesis led to the development of barrier membranes to promote selective cellular repopulation during regenerative attempts, thus promoting healing guided by the periodontal ligament and alveolar bone [1]. Various resorbable materials, such as collagen, polylactic acid and calcium sulphate, have been developed, each with unique properties and clinical considerations [2]. The study reviewed the potential applications of bovine membranes in GBR, with the objective of discussing the advantages of these membranes in the dental field, particularly in implantology, highlighting their prolonged barrier function and potential benefits over natural collagen layers [3]. Biosafety addresses concerns such as cytotoxicity, mutagenesis, carcinogenesis, while bio-functionality relates to a material's interaction with tissue, laboratory and organism-based assessments, following guidelines such as ISO 10993-3, evaluate genotoxicity, carcinogenicity and reproductive toxicity [4]. The authors evaluated the cytotoxicity and genotoxicity of bovine pericardium preserved in glycerol to assess its potential toxicity where, the unwashed pericardium was sterilized via gamma radiation and immersed in RPMI 1640 culture medium and the same extract was tested on Chinese hamster ovary cells, showing some cytotoxicity but no genotoxicity [5]. The authors evaluated the genotoxic potential of locally produced bovine pericardium using the Ames test with the exogenous metabolic activation system S9 homogenate (liver microsomal enzymes), showing no significant mutagenic effects, indicating BP membranes are safe for use in guided tissue regeneration [6]. Therefore, it is of interest to show that GTR is a dynamic and evolving field in periodontal therapy, its history, mechanisms, material types and clinical results highlight its complexity, on-going efforts to improve outcomes, biocompatibility and genetic toxicology testing are crucial for ensuring GTR's safety and effectiveness.

## Materials and Methods:

**Figure 1:**
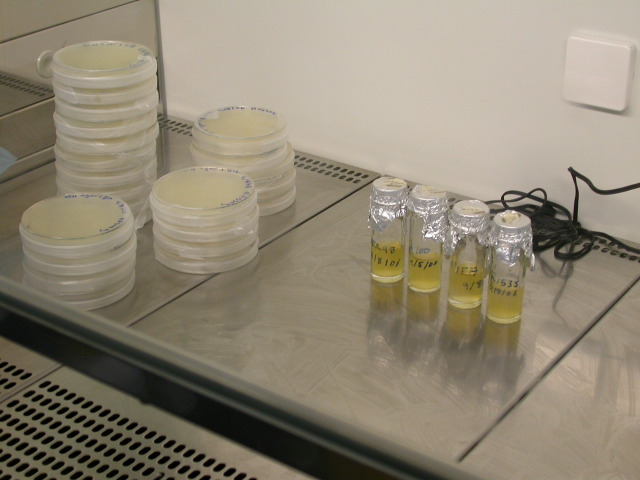
Salmonella typhimurium in nutrient broth

**Figure 2:**
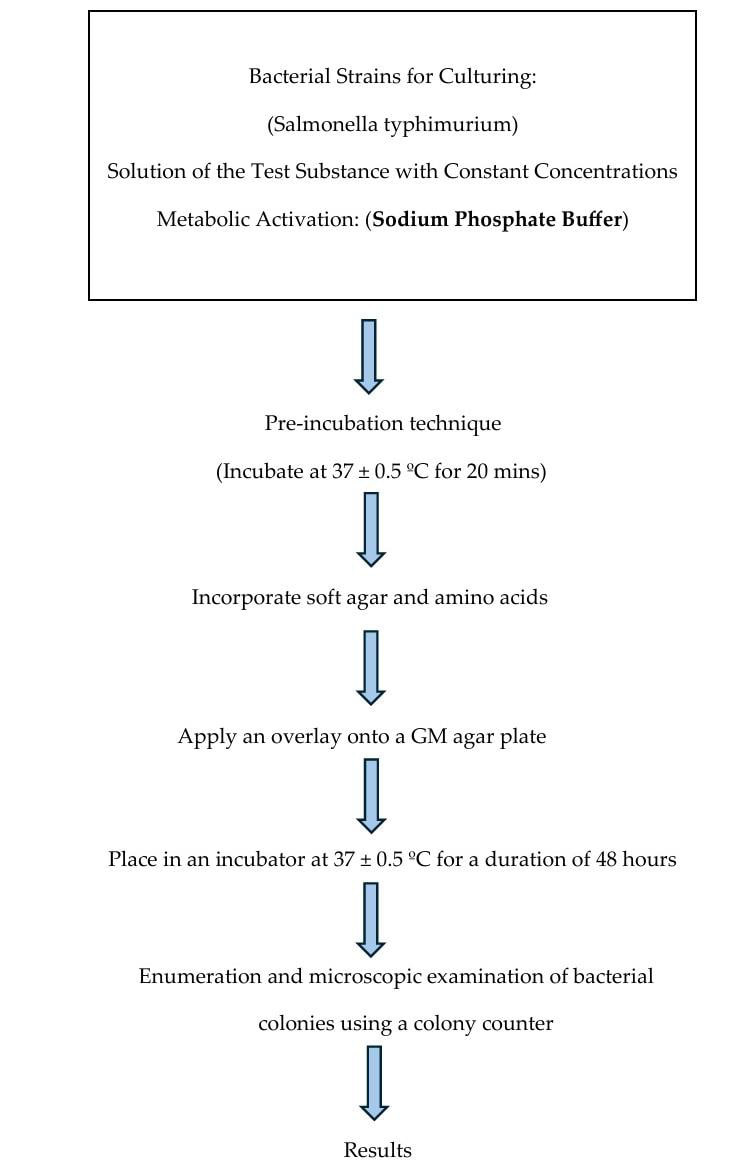
Summary of the Bacterial Reverse Mutation Test (Ames test)

The study was conducted at the Ames Test Laboratory, School of Dental Sciences and USM. The objective was to detect mutations using a bacterial reverse mutation assay influenced by the test substance, locally produced bovine pericardium (LYOLEMB), activated with a sodium phosphate buffer system. The primary evaluation criterion was counting the number of revertant colonies to assess the biocompatibility of the test substance. The tested biomaterial was locally produced bovine pericardium (LYOLEMB), primarily composed of collagen fibres, sourced from the National Tissue Bank, University Sains Malaysia, renowned for its versatility and natural properties; it was stored at room temperature under aseptic conditions. Positive controls-4-Nitro-O-phenylenediamine, sodium azide, acridine orange and 2-aminoanthracene-were sourced from various manufacturers and stored under specific conditions, then handled aseptically to ensure test accuracy. Salmonella typhimurium strains TA1535, TA1537, TA98 and TA100, used for detecting base-pair substitution and frameshift mutations, were stored at -80°C in an ultra-deep freezer (Figure 1). Each strain's characteristics included mutations affecting amino acid synthesis; DNA repair, membrane integrity and the presence of the R-factor were documented in (Table 1). Various reagents were used, including Vogel-Bonner salts, glucose solution, histidine/biotin solution, top agar, nutrient broth, sodium phosphate buffer, enriched glucose minimal agar plates, biotin and histidine solutions, ampicillin solution, crystal violet solution and nutrient agar plates. The study utilized glucose minimal agar and soft agar in the Ames test. Bovine pericardium (BP) was extracted in sterile water, incubated and then tested for mutagenicity using the standard plate incorporation assay. Positive controls were dissolved in distilled water and stored at -80°C. The Ames test was conducted using the pre-incubation method for bacterial strains TA98, TA100, TA1535 and TA1537 with sodium phosphate buffer, involving triplicates for negative controls, duplicates for test substances and positive controls. The Ames test detected genetic damages leading to mutations using a pre-incubation assay, which involved a 20-minute exposure of tester strains to the test agent, followed by plating on glucose minimal (GM) agar medium, after a 48-hour incubation at 37 ± 0.5°C, revertant colonies were counted [7] (Figure 2). Pure water was used as a negative control and various chemical agents served as positive controls for each bacterial strain (Table 2).

Microscopic examinations assessed revertant colonies and growth inhibition, with colony counts done manually or using a colony counting device, each plate was counted three times and the mean was used to determine the average number of revertant colonies per dose and the test material was deemed negative if revertant colonies were less than twice the negative control, this section details the analysis of toxicity and growth inhibition for all bacterial tester strains. This methodology offers a thorough approach to assessing bovine pericardium's genotoxicity, using standardized procedures and specific bacterial strains to ensure reliable and accurate test results, crucial for evaluating the biomaterial's safety and biocompatibility.

## Results:

The results address whether there are differences in bacterial colony counts among the strains (TA98, TA1537, TA100 and TA1535) at different test material concentrations. TA98 shows moderate variability with an average of 279.71 (range: 144.00 to 587.00). TA1537 has a higher mean of 366.86 and greater variability (range: 224.00 to 860.00). TA100 averages 350.86 with a range of 246.00 to 680.00. TA1535 has the highest mean of 415.71 and the greatest variability (range: 215.00 to 1123.00) (Table 3).

The standard deviation indicates the spread of revertant counts around the mean for each bacterial strain. TA98 and TA100 show less variability compared to TA1537 and TA1535, which have higher standard deviations. The mean revertant count varies significantly across strains, indicating a strain-specific response to the test substance and controls. The question investigates whether the number of bacterial colonies among various strains differs with variations in the concentration of the test substance and the negative control; this is determined by whether the test substance produces a bacterial count more than double that of the negative control, indicating potential toxicity. The analysis compares revertant counts in strains TA 98, TA 1537, TA 100 and TA 1535 at different concentrations of the test substance to assess toxicity.

## T-test analysis: 

The table below presents a comprehensive analysis of the test substance's effect on various bacterial strains at different concentrations, the analysis includes the mean revertant counts for the test and control, t-test results (t-statistic and p-value) and an assessment of toxicity based on whether the test mean revertant count is more than twice the control mean revertant count (Table 4). The t-statistic and p-value provide insight into the statistical significance of the difference between the test and control mean revertant counts, p-value less than 0.05 is commonly considered statistically significant. In this dataset, some comparisons show p-values close to or below this threshold, indicating a significant difference at those concentrations.

## Discussion:

We observed a 2-fold concentration-dependent increase in mean colonies for one tester strain compared to the vehicle control, Pure water served as the negative control and sodium phosphate buffer confirmed strain reliability, Positive controls included sodium azide, acridine orange, 4-nitro-o-phenylenediamine and 2-aminoanthracene,following Ames test guidelines, the highest concentration used in testing for BP was set at 5 mg/plate or 5 µl/plate, based on cytotoxicity, testing should proceed to a cytotoxic concentration if necessary and substances with significant mutagenic impurities might require testing above 5 mg/plate or 5 µl/plate and about 83% of mutagens identified also cause cancer [8]. We used the Ames test to assess the genotoxic potential of bovine pericardium (BP) membrane, a material used in guided tissue regeneration, while this rapid test is informative and requires minimal material and it uses prokaryotic cells, which differ from mammalian cells. Our study confirmed that the BP membrane has non-genotoxic potential [9]. The use of a resorbable membrane like this eliminates the need for secondary surgery, commonly used resorbable materials in both animal studies and human clinical trials include collagen, polylactic acid, polyglycolic acid and their copolymers, particularly in the management of periodontal osseous defects [10]. In evaluating the locally produced bovine pericardium (LYOLEMB) membrane as a potential biomaterial for guided tissue regeneration, we focused on its biocompatibility; it was processed with thorough cleaning, solvent dehydration and gamma irradiation sterilization. Our *in vitro* research was conducted to measure the genotoxicity of this solid membrane, which highlights its practical medical application.

We cultured bacteria to the late exponential or early stationary phase (approximately 10^9 cells per ml), avoiding late stationary phase cultures, high viable bacterial titre was crucial. We used standard bacterial reverse mutation test methods, specifically the plate incorporation and preincubation methods, with a 24-hour incubation period, as recommended by most guidelines [11]. For this study, we opted for the preincubation method; the incubation temperature was set at 37°C, approximating human body temperature, which is relevant for the bio-absorbable material of bovine pericardium used in GTR [12]. Mutagenic substances can induce reversion in histidine-deficient strains, allowing them to grow and form colonies in a histidine-limited medium, whereas non-reverted strains cannot grow, we used a set of four different strains in this study, enabling the assessment of various genomic mutations, such as frameshift mutations (TA 98 and TA 1537) and base substitutions (TA 100 and TA 1535) [13]. We used descriptive analysis and T-tests to evaluate bacterial colony counts in strains TA98, TA1537, TA100 and TA1535 at different test substance concentrations, standard deviations (TA98: 56.6, TA1537: 59.4, TA100: 39, TA1535: 61.08) showed variability, but moderate values and T-test results suggested consistent responses, maximum revertant counts did not significantly exceed means, indicating no extreme outliers, the test substance did not induce revertant counts more than twice the control level, suggesting no strong mutagenic effects and Positive controls indicated point mutations in Salmonella typhimurium, but the test substance showed no significant mutagenic activity.

The non-mutagenic properties of the LYOLEMB membrane make it a safer choice for GTR, reducing cancer risks and eliminating the need for secondary surgery. Unlike PLA and PGA, which may trigger inflammation, LYOLEMB's natural bovine origin potentially minimizes immune responses, making it a promising GTR option. Further studies are needed to confirm its benefits.

This study's findings align with research on other natural membranes like collagen, which are favoured for their low mutagenicity, biocompatibility and biodegradability, similarly, LYOLEMB's natural origin and non-inflammatory properties make it a safer alternative to synthetic materials like polylactic acid (PLA) and polyglycolic acid (PGA), which can cause inflammation during resorption [14]. Some researchers showed that bovine-derived membranes, such as Bio-Gide®, demonstrate excellent biocompatibility in periodontal applications. LYOLEMB, with its unique processing methods, could offer similar benefits while being a cost-effective, locally produced alternative [15]. The study's limitations include the absence of *in vitro* genotoxicity tests using mammalian cells, such as the micronucleus or chromosomal aberration assays, which provide a more accurate assessment of DNA damage in human tissues, while the Ames test effectively detects mutagenicity in bacteria, it may not fully predict behaviour in mammalian cells and cannot detect all genotoxic agents, particularly those causing chromosomal damage and variations in bacterial responses across strains may also affect the results, therefore, future research should focus on *in vitro* genotoxicity tests, in vivo animal studies and long-term clinical trials to confirm LYOLEMB's safety, resorption and effectiveness in GTR procedures, these studies are vital for establishing LYOLEMB as a reliable option for periodontal regeneration.

## Conclusion:

This study assessed the mutagenic potential of locally produced bovine pericardium (LYOLEMB) membranes for guided tissue regeneration using the Ames test with sodium phosphate buffer activation, revealing non mutagenic effects as revertant count did not exceed twice the negative control for any strain. These findings affirm the non-mutagenic nature of LYOLEMB, supporting its safety for clinical use in periodontal therapy. However, further research is recommended to evaluate long-term biocompatibility and compare BP membranes with other biomaterials for a comprehensive safety profile.

## Figures and Tables

**Table 1:** Characteristics of the Strains

**Strains (Salmonella typhimurium)**	**Mutation on synthesis of amino acid**	**Mutation on excision repair**	**Membrane Mutation (LPS)**	**R-Factor (PKM101)**
TA 98	hisD3052	Δ uvrB	rfa	+
TA 100	hisG46	Δ uvrB	rfa	+
TA 1535	hisG46	Δ uvrB	rfa	-
TA 1537	hisC3076	Δ uvrB	rfa	-

**Table 2:** Positive Controls of Bacterial Strains

	**TA 100**	**TA 1535**	**TA 98**	**TA 1537**
**Sodium phosphate buffer**	NaN3	NaN3	4NOP	AO
	5ug/plate	2.5ug/plate	2.5ug/plate	50 ug/plate

**Table 3:** Bacterial colony counts among the strains

**Statistical Measure**	**TA 98**	**TA 1537**	**TA 100**	**TA 1535**
Count	7	7	7	7
Mean	279.7	366.86	350.86	415.71
Standard Deviation	145	224.11	149.43	316.83
Minimum	144	224	246	215
25th Percentile	200	236	275.5	266.5
Median (50th %)	267	324	299	325
75th Percentile	280	344	340	357
Maximum	587	860	680	1123

**Table 4:** T-test analysis

**Top of Form Strain**	**Concentration**	**Mean Rev. Count (Test)**	**Mean Rev. Count (Control)**	**t-statistic**	**p-value**	**Toxicity Evaluation**
TA 98	0.3125	186	286	-2.16	0.096	No
TA 98	0.625	267	286	-0.41	0.702	No
TA 98	1.25	214	286	-1.56	0.194	No
TA 98	2.5	144	286	-3.07	0.037	No
TA 98	5	274	286	-0.26	0.808	No
TA 1537	0.3125	247	358	-2.29	0.084	No
TA 1537	0.625	225	358	-2.74	0.052	No
TA 1537	1.25	324	358	-0.7	0.522	No
TA 1537	2.5	224	358	-2.76	0.051	No
TA 1537	5	330	358	-0.58	0.595	No
TA 100	0.3125	246	352	-3.33	0.029	No
TA 100	0.625	269	352	-2.61	0.06	No
TA 100	1.25	299	352	-1.67	0.171	No
TA 100	2.5	328	352	-0.75	0.493	No
TA 100	5	282	352	-2.2	0.093	No
TA 1535	0.3125	382	287	1.9	0.13	No
TA 1535	0.625	215	287	-1.44	0.222	No
TA 1535	1.25	325	287	0.76	0.489	No
TA 1535	2.5	246	287	-0.82	0.457	No
TA 1535	5	332	287	0.9	0.418	No
